# Prospective phase II study of preemptive chimerism-driven reduction of immunosuppression after non-myeloablative conditioning—Eudract #: 2007-002420-15

**DOI:** 10.1038/s41409-022-01609-6

**Published:** 2022-02-18

**Authors:** Saskia Hell, Madlen Jentzsch, Georg-Nikolaus Franke, Nadja Jäkel, Susann Schulze, Jeanett Edelmann, Kolja Nenoff, Nora Grieb, Veljko Jeremic, Michael Cross, Sabine Leiblein, Enrica Bach, Wolfram Pönisch, Haifa-Kathrin Al-Ali, Sebastian Schwind, Uwe Platzbecker, Thoralf Lange, Dietger Niederwieser, Vladan Vucinic

**Affiliations:** 1 Leipzig Medical Center, Medical Clinic and Policlinic 1, Hematology, Cellular Therapy and Hemostaseology, University Leipzig, Leipzig, Germany; 2 Department of Hematology/Oncology, University Hospital Halle, Halle (Saale), Germany; 3 Krukenberg Cancer Center, University Halle, Halle (Saale), Germany; 4 Leipzig Medical Center, Institute of Legal Medicine, University Leipzig, Leipzig, Germany; 5 Department for Operations Research and Statistics, Faculty of Organizational Sciences, University of Belgrade, Belgrade, Serbia

**Keywords:** Stem-cell therapies, Haematological cancer

## To the Editor:

Allogenic stem cell transplantation (alloHSCT) is one of the most effective curative therapies for hematologic malignancies. In order to minimize the required cytotoxic dose, reduced intensity and non-myeloablative regimens (NMA) have been developed and today render alloHSCT accessible to frail and comorbid patients [1, 2].

NMA conditioning regimens are based on donor engraftment without eradication of host hematopoesis with induction of a complete donor chimerism as the goal [3]. The successive careful monitoring of the dynamics of chimerism is necessary to predict hematological relapse and to schedule preemptive therapeutic interventions in such patients [4].

The sensitivity and prognostic relevance of donor chimerism may be increased by focusing specifically on the lineages affected by the underlying hematologic neoplasm. Since a decrease in this lineage specific chimerism (LSC) often occurs prior to hematological relapse, LSC may be an even more sensitive and valuable early predictor of relapse than the complete chimerism.

We performed a prospective, open-labeled, single-center, phase II trial with the aim to demonstrate the efficacy of an early reduction of immunosuppression in patients with hematologic malignancies experiencing a decrease in LSC after alloHSCT. The primary objective was to determine the incidence of hematologic relapse after early reduction of immunosuppression following a decrease of >10% LSC in patients with previously full (≥90%) LSC. Secondary objectives were the reconversion of LSC after reduction of immunosuppression as well as the incidence of graft versus host disease (GvHD).

Between May 2008 and July 2012, a total of 200 patients at a median age of 61.9 (range 26.6–74.7) years at alloHSCT were included in the trial. Of those, 154 patients showed LSC ≧90% and had sufficient follow-up data available to be included in the outcome analysis. Patients suffered from a malignant hematologic disease with an indication for alloHSCT and were unfit for myeloablative conditioning due to higher age or comorbidities.

The demographic data, conditioning regimen, the tapering of immunosuppression, details on chimerism analysis as well as subanalyses of patients with myeloid or non-myeloid neoplasms are presented in the Supplementary Material.

The median follow-up after alloHSCT was 3 years.

No decrease in LSC was observed in 133 (67.9%) patients during further follow-up. 13 (9.7%) of them developed hematologic relapse without LSC decrease ≥10% while 43 (32.3%) patients suffered NRM.

Twenty-one (10.7%) patients (15 AML, 3 MDS, 1 MM, 1 CML, 1 CLL) showed a decrease ≥10% in LSC during the first year after alloHSCT (median 71 days, range 48–357 days) without immediate hematologic relapse. The reduction of immunosuppression according to the protocol was possible in all but one patient (95%) who developed signs of acute skin GvHD. After the reduction of immunosuppression, LSC increased to ≧90% in 7/20 patients (35.0%), three of whom relapsed with simultaneous LSC decrease later-on.

Four patients showed a complete chimerism without hematologic relapse. Two of these four patients showed no sign of GvHD at any time, both are still alive and in remission. One patient developed relapse 117 days after IS reduction and a GvHD grade 1 of skin simultaneously, whereas the fourth patient developed a GvHD of the liver grade 4 and showed relapse with repeated decrease in LSC 369 days after the reduction of immunosuppression.

All the patients who reconverted the LSC after reduction of immunosuppression had AML (75%) or MDS (25%) as underlying diseases.

Eleven patients with LSC decrease ≥10% relapsed regardless of reduction in immunosuppression after a median of 84 days (six simultaneously with decrease of chimerism, five patients after median 30 [range 19–83] days after decrease of LSC). Two more patients presented further decrease of LSC and died from cardiac arrest and pneumonia, one patient died from intestinal GvHD with no sign of relapse.

Overall, 13 of 20 patients (65%) developed an acute GvHD after reduction of immunosuppression and five of them showed signs of GvHD grade 3 and 4 (one hepatic, four intestinal).

**Fig. 1:**
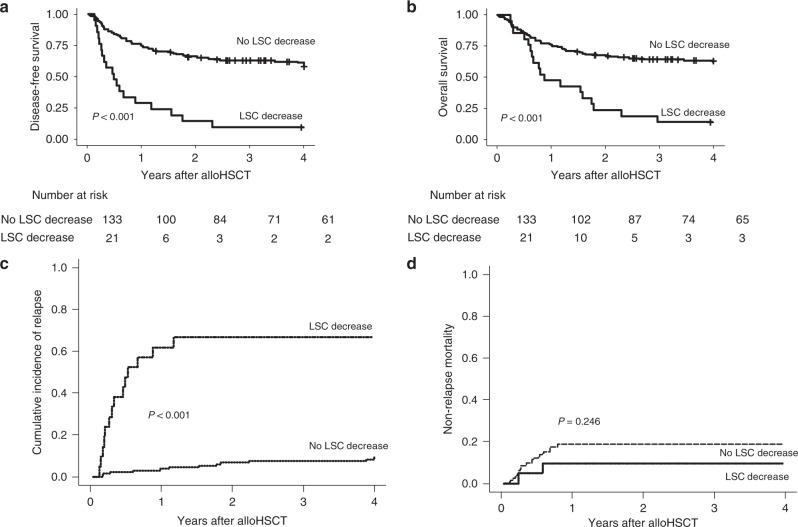
Outcome of the whole patient cohort according to decrease in lineage specific chimerims (LSC) (*n* = 154). **a** Disease-free survival, **b** Overall-survival, **c** Cumulative incidence of relapse, **d** Non-relapse mortality.

The outcomes of the patients according the LSC decrease are presented in Fig. 1.

Our prospective open-labeled study analyzed the role of bone marrow LSC as a follow-up parameter in terms of engraftment and relapse prevention after alloHSCT with NMA conditioning. We could show that early reduction of immunosuppression could increase the LSC and subsequently prevent hematologic relapse in 20% of patients.

The efficient management of patients following NMA alloHSCT requires close monitoring of donor chimerism and adjustment of immunosuppression as necessary to maintain donor chimerism and the graft-vs.-tumor effect while avoiding reemergence of the leukemic clone or debilitating GvHD [5]. The current Criteria of American Society of Transplantation and Cellular Therapy recommend the determination of chimerism in the clinical routine on day +30, +90, +180 and 1 year after alloHSCT [6].

Bornäuser et al. showed that a decrease of CD34^+^ donor chimerism to levels <80% leads to hematologic relapse within a median time of 61 days, thus making further intervention necessary [7].

In our study 5/20 patients developed a severe GvHD (grade ≥3) after reduction of immunosuppression, limiting them for further interventions, especially donor lymphocyte infusions (DLI).

The treatment with DLI may induce remission rates ranging from 20 to 80% in patients with hematologic relapse after alloHSCT for AML or CML [8]. However, DLI cannot be applied in patients with GvHD or in those whose donors are not willing or eligible for successive DLI donation. Furthermore, the time needed to produce DLI can be a limiting factor for imminent treatment, further underlining the utility of LSC-triggered reduction of immunosuppression.

Alternatively, in AML and MDS patients with decrease of CD34+ chimerism in peripheral blood to <80% and absence of hematological relapse, the application of 5-azacitidine was able to prevent relapse in 80% patients [9].

The use of LSC as measurable residual disease (MRD) marker for post-transplant monitoring can lead to even earlier interventions with the possibility of better outcomes. In the recently published RELAZA2 trial of 60 patients who developed MRD 24 months after alloHSCT, 46% were relapse free and alive 12 months after application of 5-azacitidine [10]. This raises the question if it is possible to identify the population of patients who can be rescued from imminent hematological relapse without the use of hypomethylating therapies especially if DLI are not available.

Furthermore, many other factors implicate an early relapse. Our group emphasized the prognostic value of Wilms Tumor gene 1 (*WT1*) in combination with CD34+ bone marrow chimerism in a retrospective analysis of 88 transplanted patients with AML and MDS. CD34+ sorted LSC together with the assessment of *WT1* transcripts from peripheral blood can provide an accurate indication of impending relapse [11]. Rautenberg et al. reported on negative prognostic impact of WT1 status prior to alloHSCT [12]. Unfortunately the determinations of *WT1* are complex, expensive and not universally applicable tools.

In this trial we evaluated the chimerism-driven early reduction or termination of immunosuppression prior to development of hematologic relapse in patients undergoing alloHSCT after NMA conditioning. Limited to the intervention on only 20 patients, we conclude that the reduction of immunosuppression is safe and was able to prevent the hematological relapse in 20% of cases. However, this approach needs to be combined with other interventions like DLI, hypomethylating agents, or targeted therapies.

## Supplementary information

Supplemental Material

SM Figure 1

SM Figure 2

SM Figure 3

SM Figure 4
