# Supplementary material for: Prospective phase II study of preemptive chimerism-driven reduction of immunosuppression after non-myeloablative conditioning—Eudract #: 2007-002420-15
Source: Bone Marrow Transplant. 2022 Feb 18;57(5):824–6. doi: 10.1038/s41409-022-01609-6 (PMC9090627; doi:10.1038/s41409-022-01609-6)
Supplement: Supplementary file 1 — Supplemental Material [file 41409_2022_1609_MOESM1_ESM.docx]

**Supplemental material:**

**Prospective Phase II Study of preemptive Chimerism-driven Reduction of Immunosuppression after Non-Myeloablative Conditioning - Eudract #: 2007-002420-15**

Hell *et al.*

1. **Study design and endpoints**

This is a prospective, open-labeled, single-center, phase II trial performed at the University Leipzig, Leipzig Medical Center in Germany. The study was conducted according to the Declaration of Helsinki and received the approval of an Institutional Review Board by the Ethical Board of the University of Leipzig. The trial was registered at European clinical trials database (Eudract#:**2007-002420-15**). Written informed contest was obtained from all patients.

The primary objective of this study was to determine the incidence of hematologic relapse after early reduction of immunosuppression following a decrease of > 10% donor line specific chimerism (LSC) in patients with previously full ($\geq$ 90%) LSC.

Secondary objectives were the reconversion of LSC after reduction of immunosuppression as well as the incidence of graft versus host disease (GvHD).

1. **Patients and treatment**

Between May 2008 and July 2012, a total of 200 patients at a median age of 61 (range 26 -74) years at alloHSCT were included in the trial. Of those, 154 patients showed LSC ≥ 90% and had sufficient follow-up data available to be included in the outcome analysis. 42 patients did not show LSC ≥ 90% on day 28 and/or 56 and were excluded from the analysis. Four patients were lost to follow up and were also excluded from analysis (SM Figure 1).

1. **Inclusion and exclusion criteria of the trial**

Inclusion criteria:

- male and female patients not available for allogeneic hematopoetic stem-cell transplantation (alloHSCT) with myeloablative conditioning

- Indication for alloHSCT^1^

- Karnofsky Index ≥ 70%

- negative pregnancy test in female patients

- written patient content

Exclusion criteria:

- Active solid tumor
- Pregnant or brest-feeding female patients
- Patients with allergy or intolerance against mycophenolacid, mycophenolatmofetil or against Cyclosporin-A
- Patients unwilling to perform effective contraception
- Patients with fungal pneumonia, progredient after four-week treatment with amphotericine-B derivates or other substances against aspergillus
- Reduction of following organ functions:
  - Creatinine-clearance<50 ml/min/1.73m^2^
  - Severe pulmonary function reduction
  - Bilirubine > 2 x upper normal level (ULN), transaminases > 4x ULN
  - Infection with human immunodeficiency virus

1. **Trial procedure**

The trial procedure is depicted in the consort diagram (SM Figure 1).

1. **Demographic characteristics of the patients**

The demographic characteristics, the graft source and underlying diseases are presented in the Table 1.

1. **Conditioning regimen**

Prior to allogeneic hematopoietic stem cell transplantation (alloHSCT) all patients received non-myeloablative (NMA) conditioning regimen consisting of fludarabine 30mg/m2 on day -4 through -2 and a 200 cGy total body irradiation on day 0.^2^ Antithymocyte globulin (ATG) was not part of the conditioning, and the grafts were not T-cell depleted. The data regarding donor source are presented in supplemental material (SM Table 1).

1. **Immunosuppression and Graft-vs-host disease (GvHD)**

All patients received immunosuppression with cyclosporine A (CSA) starting from day -1 and was adjusted to a target blood level of 200 ng/ml. Additionally, patients received mycophenolate mofetil (MMF) starting from day 0 eight hours after alloHSCT at a dosage of 1000 mg BID after alloHSCT from matched related donor (MRD) or 1000 mg three times a day after alloHSCT from matched unrelated donor (MUD) or mismatched unrelated donor (MMUD) on allele or antigene level. In the absence of apparent acute GvHD, the prophylaxis with MMF was discontinued at day 28 after alloHSCT from MRD or reduced by 500 mg every two weeks starting on day 40 after alloHSCT from MUD or MMUD. In the absence of a GvHD, CSA was reduced by 25 mg weekly from day 84 after MRD HSCT and from day 180 after alloHSCT from a MUD and discontinued by day 270 in all patients.

1. **Chimerism analyses**

Lineage specific chimerims (LSC) analyses were performed on flow sorted cells from bone marrow aspirate using fluorescent *in situ* hybridization (XY-FISH) for gender variant donors and by short tandem repeats as previously reported^3^ for gender identical donors on day + 28, +56, +84, +180 and every 6 months thereafter, as previously described.^4^ In case of reduction of immunosuppression for decreasing LSC without hematologic relapse, subsequent bone marrow examinations were performed after 4 to 8 weeks.

We used CD34^+^ for patients with acute myeloid leukemia, myelodysplastic syndromes, and myeloproliferative neoplasia including chronic myeloid leukemia, CD19^+^ for chronic lymphocytic leukemia and B-non-hodgkin lymphoma and CD138^+^ for multiple myeloma patients. The patients with T-non-hodgkin lymphoma were monitored by the CD3^+^ chimerism.

1. **LSC-adjusted early reduction of immunosuppression**

In patients with a decrease of LSC without signs of hematologic relapse, MMF was reduced weekly by 50% as long as the daily dose was > 1500 mg and discontinued when the dose dropped below 1500 mg. CSA dose was reduced by 50% when dose levels were above 150 ng/ml, by 75% for dose levels between 100 and 150 ng/ml and discontinued from dose levels below 100 ng/ml. The occurrence of acute GvHD was assessed according to Glucksberg criteria.^5^ The patients with acute GvHD grade > 2 were treated with corticosteroids according to the institutional standards.

1. **Statistical analysis**

Overall survival (OS) was defined as the probability of survival, regardless of disease status, from the time of alloHSCT. Surviving patients were censored at last follow-up and only death was considered an event. Disease free survival (DFS) was defined as the probability of survival, without evidence of malignancy after alloHSCT. In estimating DFS, death and relapse were considered events. Non-relapse-mortality (NRM) was defined as the probability of death from any cause other than recurrence of the disease after alloHSCT. The patients underwent the follow-up for four years.

The probability of DFS and OS was assessed according to the Kaplan-Meier estimate. NRM and cumulative incidence of relapse (CIR) were considered to be reciprocal competing risks and were calculated according to the Fine and Gray model.^6^ Curve comparisons and p values were analysed using the R project (version 3.6.2).

1. **Outcome of the whole population**

SM Figure 2

1. **Outcome of patients with myeloid neoplasm**

SM Figure 3

1. **Outcome of patients with non-myeloid diseases**

SM Figure 4

1. **Patients without LSC reconstitution 28 and/or 56 days after alloHSCT**

42 (21.4%) patients did not show LSC ≥ 90% in bone marrow assessments on days +28 and +56. In 36 (85.7%) of them relapse or persistence of hematological disease was detected on day +28 or +56. Two patients died on infections without detection of hematological relapse on days 127 and 132 respectively. One patient showed a transplant rejection and underwent a subsequent transplantation after one month. Three other patients with chronic lymphocytic leukemia developed full LSC after 353, 442 and 861 days after alloHSCT respectively.

**Table 1:** *Overview on characteristics of evaluable patients (n=196).*

|  | **Total population** | **LSC decrease** | **no-LSC decrease** | **no LSC reconstitution d+28 and/or d+56** |
| --- | --- | --- | --- | --- |
| **Characteristics (n)** | 196 | 21 | 133 | 42 |
| sex, n (%) |  |  |  |  |
| male | 126 (64.9) | 12 (52.2) | 88 | 26 (61.9) |
| female | 70 (35.1) | 9 (42.8) | 45 | 16 (38.1) |
| median age (range) at alloHSCT in years | 61.9 (26.6 – 74.7) | 66.7 (46.8 –70.9) | 61.1 (26.1 – 73.9) | 65.4 (32.2 –74.7) |
|  |  |  |  |  |
| **Disease, n (%)** |  |  |  |  |
| AML | 86 (43.8) | 15 (71.4) | 50 (37.6) | 21 (50.0) |
| MM | 27 (13.8) | 1 (4.8) | 21 (15.7) | 5 (11.9) |
| MDS | 27 (13.8) | 4 (19.0) | 17 (12.8) | 5 (11.9) |
| NHL | 38 (19.4) | 0 (0.0) | 30 (22.6) | 8 (19.0) |
| ALL | 7 (3.6) | 0 (0.0) | 7 (5.3) | 0 |
| MPN | 11 (5.6) | 1 (4.8) | 8 (6.0) | 3 (7.1) |
|  |  |  |  |  |
| **Donor** |  |  |  |  |
| MRD, n (%) | 25 (12.7) | 5 (23.8) | 18 (13.5) | 2 (4.8) |
| MUD, n (%) | 126 (64.3) | 11 (52.4) | 85 (63.9) | 30 (71.4) |
| MMUD, n, (%) | 45 (23.0) | 5 (23.8) | 30 (22.6) | 10 (23.8) |

Legend: ALL – acute lymphoblastic leukemia, AML – acute myeloid leukemia, d – day, LSC – line specific chimerism, MDS – myelodysplastic syndrome, MMUD – mismatched unrelated donor, MPN – myeloproliferative neoplasia, MRD – matched related donor, MUD – matched unrelated donor

**SM Figure 1:** Consort diagram of patients’ treatment population

**SM Figure 2:** Outcome of the whole population (n=154)

**SM Figure 3:** Outcome of patients with myeloid neoplasms according to decrease in LSC (n=94)

Legend: LSC – line specific chimerism

**SM Figure 4:** Outcome of patients with non-myeloid diseases (n=60)

Legend: LSC – line specific chimerism

**References:**

1 Ljungman P, Urbano-Ispizua A, Cavazzana-Calvo M, Demirer T, Dini G, Einsele H *et al.* Allogeneic and autologous transplantation for haematological diseases, solid tumours and immune disorders: Definitions and current practice in Europe. *Bone Marrow Transplant* 2006; **37**: 439–449.

2 McSweeney PA, Niederwieser D, Shizuru JA, Sandmaier BM, Molina AJ, Maloney DG *et al.* Hematopoietic cell transplantation in older patients with hematologic malignancies: Replacing high-dose cytotoxic therapy with graft-versus-tumor effects. *Blood* 2001; **97**: 3390–3400.

3 Antin JH, Childs R, Filipovich AH, Giralt S, Mackinnon S, Spitzer T *et al.* Establishment of complete and mixed donor chimerism after allogeneic lymphohematopoietic transplantation: Recommendations from a workshop at the 2001 Tandem Meetings of the International Bone Marrow Transplant Registry and the American Society of Blood an. *Biol Blood Marrow Transplant* 2001; **7**: 473–485.

4 Maris MB, Niederwieser D, Sandmaier BM, Storer B, Stuart M, Maloney D *et al.* HLA-matched unrelated donor hematopoietic cell transplantation after nonmyeloablative conditioning for patients with hematologic malignancies. *Blood* 2003; **102**: 2021–2030.

5 Glucksberg H, Storb R, Fefer A, Buckner CD, Neiman PE, Clift RA *et al.* Clinical manifestations of graft-versus-host disease in human recipients of marrow from hl-a-matched sibling donors1. *Transplantation* 1974; **18**: 295–304.

6 Fine JP, Gray RJ. A Proportional Hazards Model for the Subdistribution of a Competing Risk. *J Am Stat Assoc* 1999; **94**: 496–509.
