# Supplementary figures and images for: Prospective phase II study of preemptive chimerism-driven reduction of immunosuppression after non-myeloablative conditioning—Eudract #: 2007-002420-15
Source: Bone Marrow Transplant. 2022 Feb 18;57(5):824–6. doi: 10.1038/s41409-022-01609-6 (PMC9090627; doi:10.1038/s41409-022-01609-6)

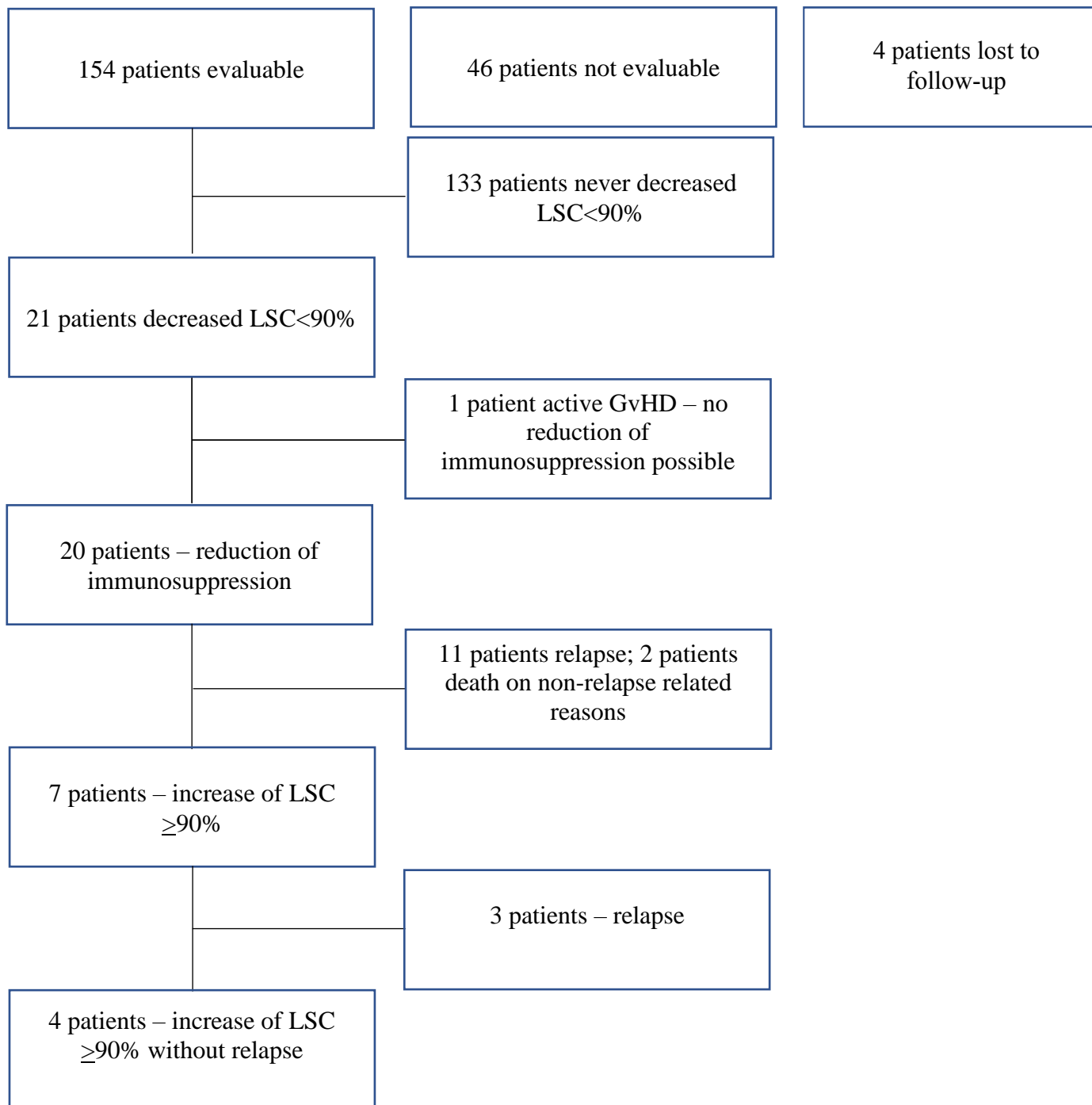

Supplement: Supplementary file 2 — SM Figure 1 [file 41409_2022_1609_MOESM2_ESM.pdf]

**A**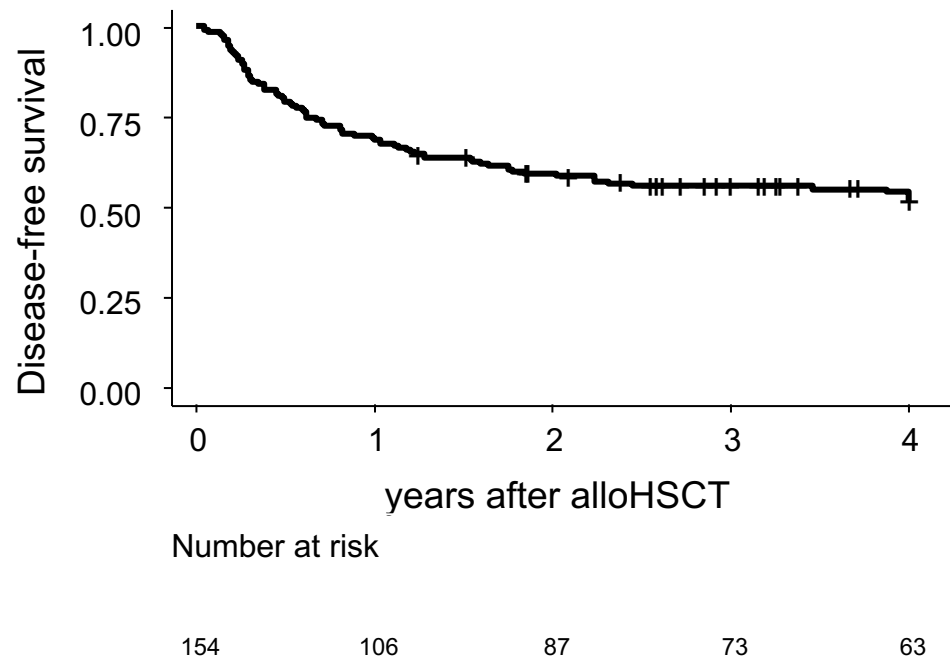**B**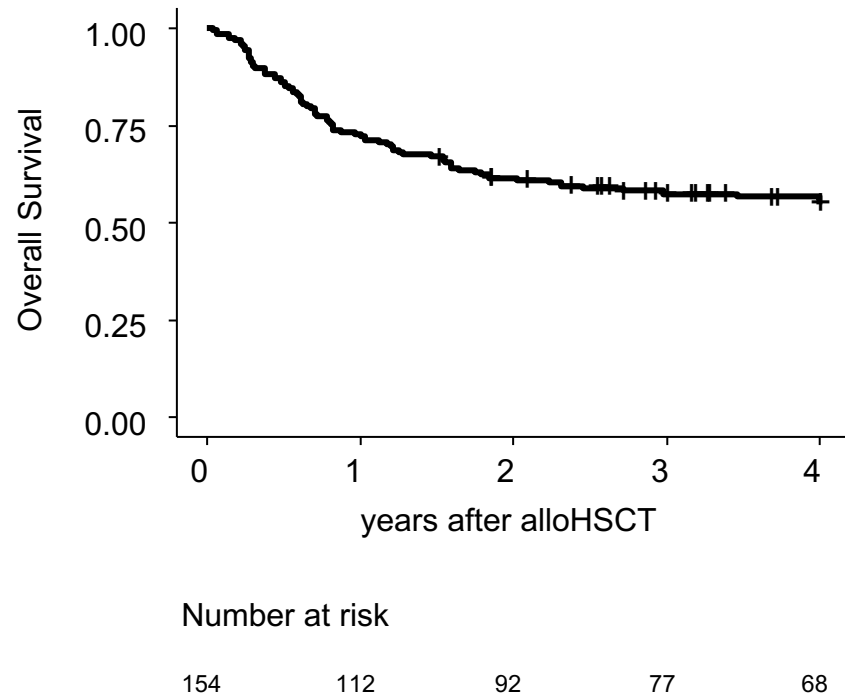

Supplement: Supplementary file 3 — SM Figure 2 [file 41409_2022_1609_MOESM3_ESM.pdf]

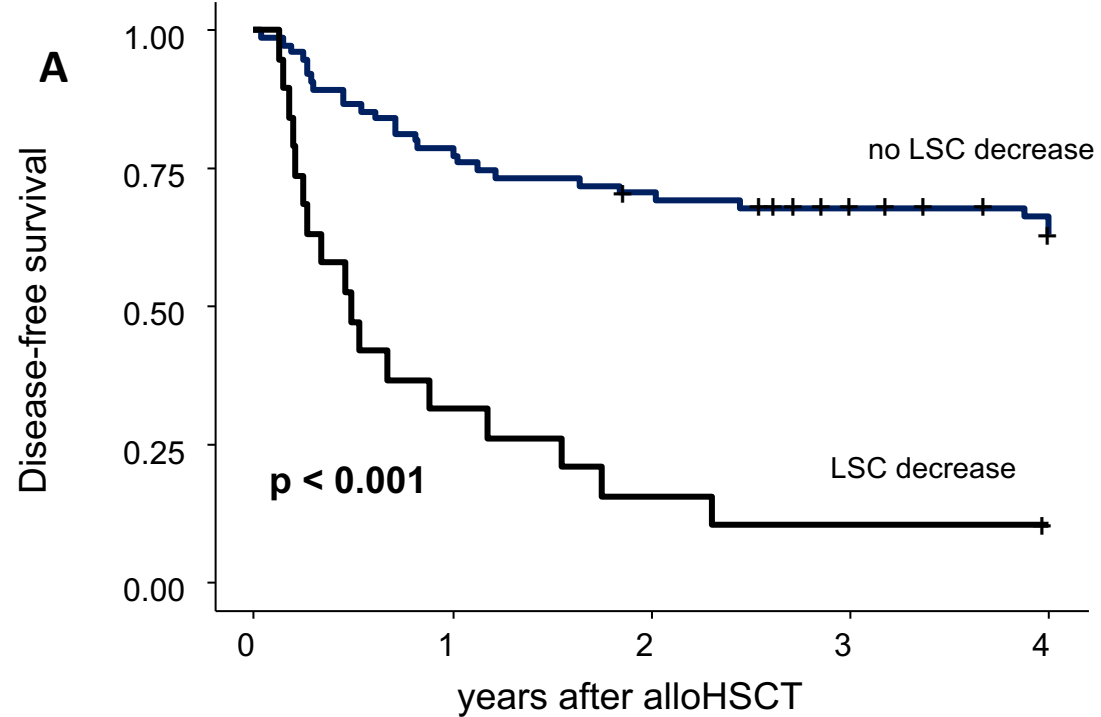

Number at risk

|                 |    |    |    |    |    |
|-----------------|----|----|----|----|----|
| LSC decrease    | 75 | 59 | 52 | 45 | 40 |
| no LSC decrease | 19 | 6  | 3  | 2  | 2  |

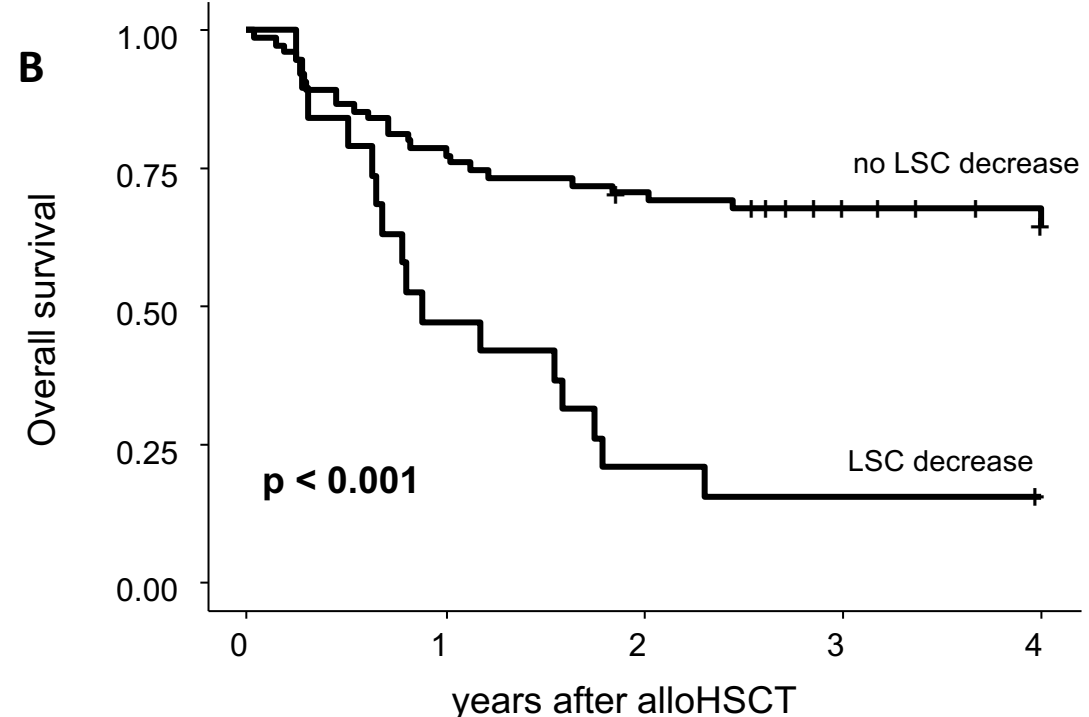

Number at risk

|                 |    |    |    |    |    |
|-----------------|----|----|----|----|----|
| no LSC decrease | 75 | 59 | 52 | 45 | 41 |
| LSC decrease    | 19 | 9  | 4  | 3  | 3  |

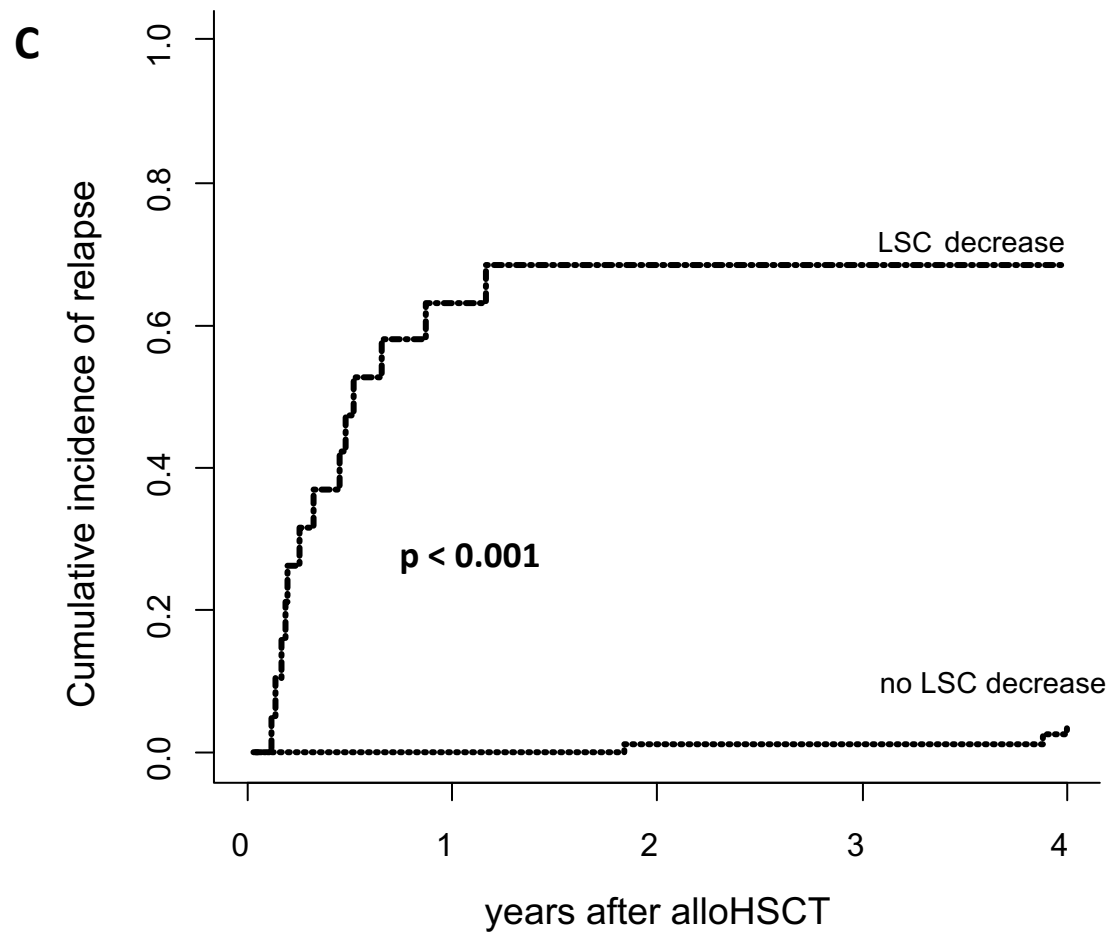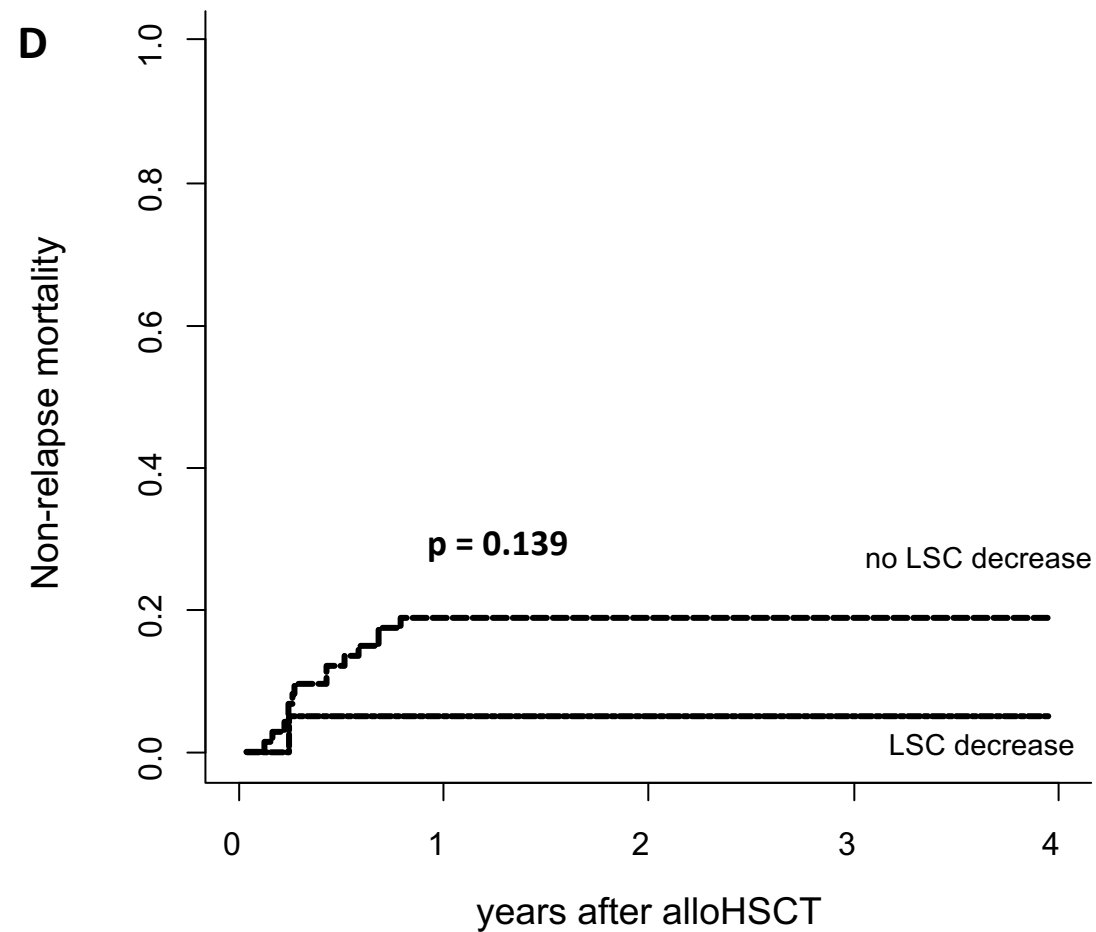

Supplement: Supplementary file 4 — SM Figure 3 [file 41409_2022_1609_MOESM4_ESM.pdf]

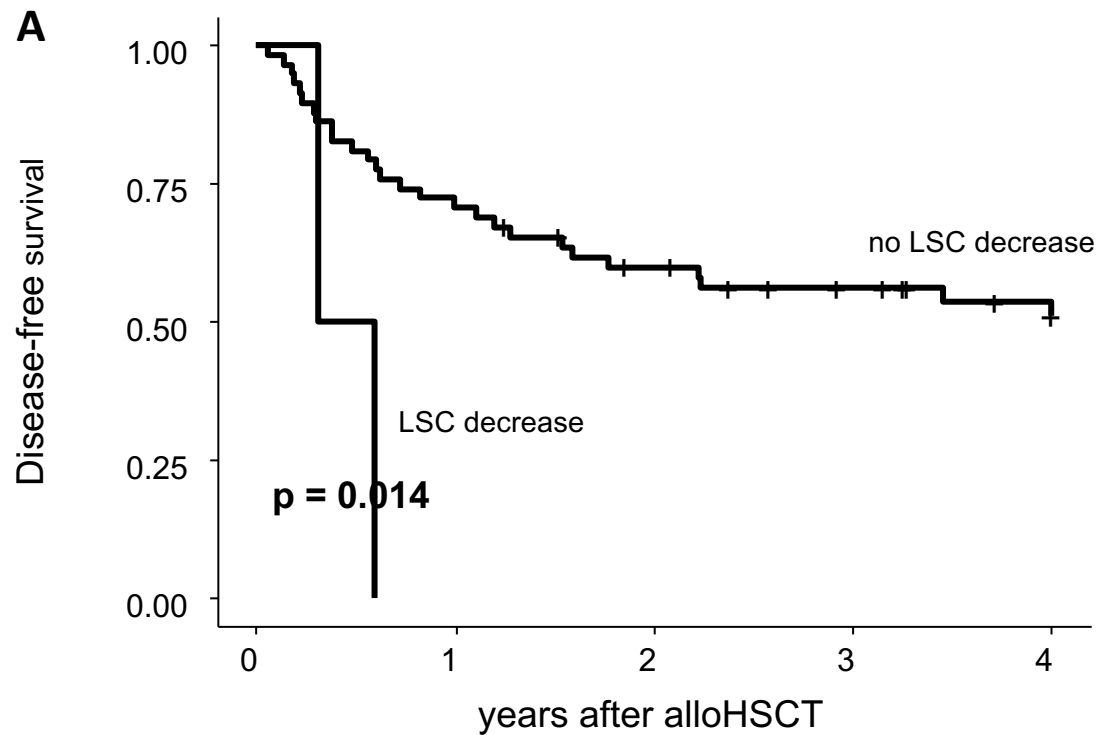

Number at risk

|                 |    |    |    |    |    |
|-----------------|----|----|----|----|----|
| no LSC decrease | 58 | 41 | 32 | 26 | 21 |
| LSC decrease    | 2  | 0  | 0  | 0  | 0  |

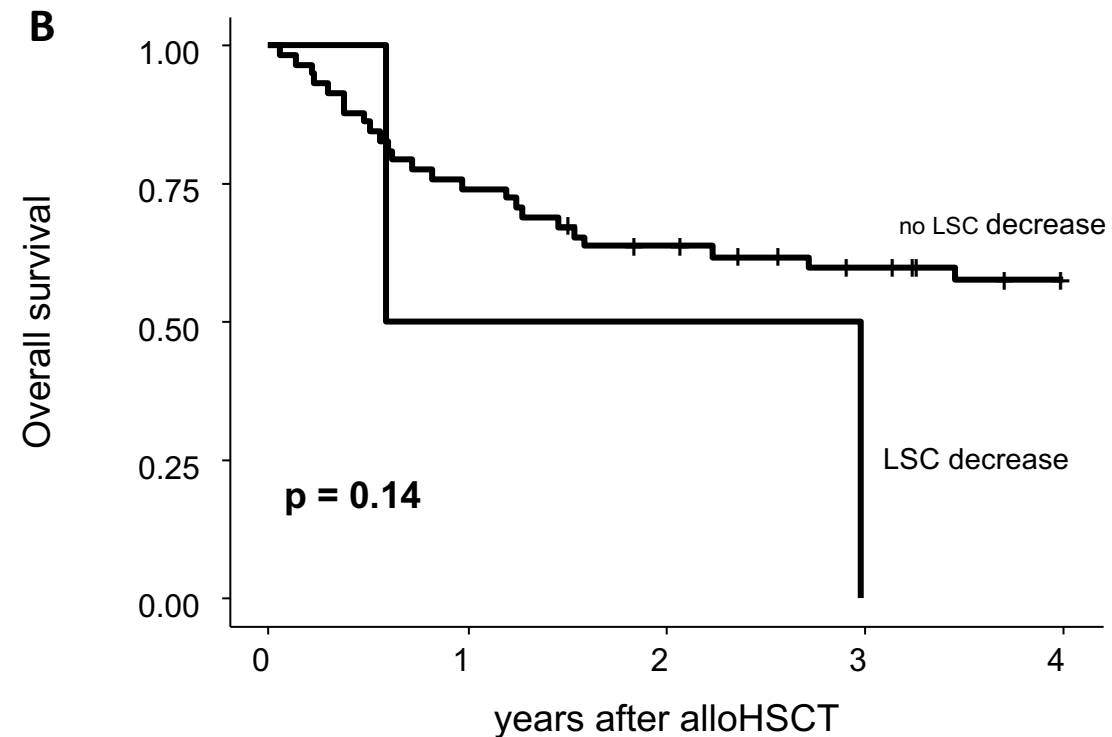

Number at risk

|                 |    |    |    |    |    |
|-----------------|----|----|----|----|----|
| no LSC decrease | 58 | 43 | 35 | 29 | 24 |
| LSC decrease    | 2  | 1  | 1  | 0  | 0  |

Supplement: Supplementary file 5 — SM Figure 4 [file 41409_2022_1609_MOESM5_ESM.pdf]
